# High prevalence and antimicrobial susceptibility pattern of *salmonella* species and extended-spectrum β-lactamase producing *Escherichia coli* from raw cattle meat at butcher houses in Hawassa city, Sidama regional state, Ethiopia

**DOI:** 10.1371/journal.pone.0262308

**Published:** 2022-01-14

**Authors:** Woyinshet Worku, Moges Desta, Tadesse Menjetta

**Affiliations:** 1 Hawassa College of Health Sciences, Department of Medical Laboratory Science, Hawassa, Ethiopia; 2 Hawassa University, College of Medicine and Health Sciences, School of Medical Laboratory Science, Hawassa, Ethiopia; Suez Canal University, EGYPT

## Abstract

**Background:**

Food-borne diseases related to the consumption of meat and its products had public health importance worldwide. The problem became worst in Ethiopia as the result of the tradition of eating raw cattle meat. *Salmonella* species and *Escherichia coli* are important food-borne pathogens associated with meat contamination. Hence the current study aimed to assess the prevalence and antimicrobial susceptibility of *Salmonella* species and Extended-spectrum β-lactamase producing *Escherichia coli* from raw cattle meat at butcher houses in Hawassa city, Sidama regional state, Ethiopia.

**Method:**

A cross-sectional study was done on the prevalence and antimicrobial susceptibility pattern of *Salmonella* species and Extended-spectrum β-lactamase producing *E*.*coli* from raw cattle meat at butcher houses in Hawassa city from September to December 2020. Socio-demographic data were collected using a structured questionnaire and raw cattle meat and swab samples were collected from meat cutting equipment. The collected samples transported using icebox to Hawassa University College of Medicine and Health Sciences Microbiology Laboratory for identification. Samples were grown on different culture media and antimicrobial susceptibility tests were determined by using Kirby disc diffusion method. Data were entered and analyzed into SPSS version 23. Descriptive statistics were done and P-value < 0.05 was considered as statistically significant.

**Result:**

The overall prevalence of *salmonella* and ESBL producing *E*.*coli* among 556 samples collected from 278 butcher houses was 36 (6.47%) (95% CI: 1.68–1.79) of which 13 (2.3%) were ESBL producing *E*.*coli* and 23(4.1%) were *salmonella* species. Poor hand washing practice (AOR = 2.208; 95% CI: 1.249–3.904) and touching birr while selling meat (AOR = 0.75; 95% CI: (0.433–1.299) were found to be significantly associated with the prevalence of *salmonella s*pecies and *E*.*coli* on cattle meat. The isolates showed moderate levels of resistance (60–70%) against Amoxicillin/ clavulanic acid and high susceptibility (85–100%) against gentamicin, cotrimoxazole, ceftazidime, and tetracycline and the overall multidrug resistance was 33.3%.

**Conclusion:**

This study revealed moderately high prevalence of *salmonella* and *E*.*coli* due to poor hygiene and sanitation practices in the butcher shops. Furthermore, the existence of ESBL producing *E*.*coli* isolates clearly indicate the possible threat to public health. Therefore, inspection by the right agencies must be implemented in order to prevent food-borne outbreaks and antimicrobial resistance.

## Introduction

Meat is the main source of protein and valuable qualities of vitamins for most people in various parts of the world and is essential for the growth, repair, and maintenance of body cells and crucial for our everyday activities [1]. Due to this rich composition, it offers a highly favorable environment for the growth of pathogenic bacteria [2]. Food-borne microorganisms are major pathogens affecting food safety and cause human disease worldwide as a result of consumption of foodstuff, mainly animal products contaminated with vegetative pathogens or their toxins [3].

The majority of food-borne bacteria frequently cause self-limiting gastroenteritis but, invasive diseases and various complexities may also occur. Among these, *E*. *coli* causes bloody diarrhea and hemolytic uremic syndrome and *Salmonella* causes systemic salmonellosis [4]. *Salmonella* has been the problem of public health concern as an agent causing food-borne diseases for over a century. It has been estimated to be responsible for 30% of the food-borne outbreaks in the United States [5]. In 2015, the World Health Organization (WHO) Food-borne Disease Burden Epidemiology Reference Group published the world’s first estimates of the global and regional incidence and burden of food-borne disease (FBD). It is estimated that in 2010, 31 major food-borne hazards resulted in over 600 million illnesses and 420,000 deaths worldwide in 2010 [6].

*Salmonella* has been the problem of public health concern as an agent causing food-borne diseases for over a century. It has been estimated to be responsible for 30% of the food-borne outbreaks in the United States [5]. Salmonellosis is a common food borne disease and is caused by a very diverse group of *Salmonella enterica* strains. The major pathogenic serovars of *Salmonella enterica* that infect humans from a variety of different food products include *Salmonella* e*nteritidis* and *Salmonella typhimurium* [7].

*E*. *coli* is a natural inhabitant of the human intestinal tract and warm-blooded animals and it used as an indicator bacterium since these bacteria acquires antimicrobial resistance faster than other conventional bacteria. *E*.*coli* is a well-known commensal of the gastrointestinal tract of vertebrates, including humans, but it is also involved in intestinal and extra intestinal pathologies [8].

Extended-spectrum β-lactamase (ESBL) producing Gram negative bacteria are considered as the main health problem, globally. The beta lactam rings of third generation cephalosporins are hydrolyzed by the ESBL enzyme which alters the structure of the antibiotic. Due to the alteration in the structure of the antibiotic, bacteria show resistance to these antibiotics [9]. ESBLs-producing *E*.*coli* isolates have emerged as a global threat to human health, and have been isolated from human, animal and environmental origins. The small but progressively increasing use of third generation cephalosporins in food animal production may be associated with the recent emergence of ESBLs-producing bacteria that are related to cattle [10].

Multidrug resistance has been increased all over the world that is considered a public health threat. Several recent investigations reported the emergence of multidrug-resistant bacterial pathogens from different origins including humans, birds, cattle, and fish that increase the need for routine application of the antimicrobial susceptibility testing to detect the antibiotic of choice as well as the screening of the emerging MDR strains [11].

The occurrence of MDR has increased worldwide. The MDR bacterial pathogens from several origins were described by numerous recent studies that reflect public health threats [12, 13]. Continuous surveillance of antimicrobial susceptibility is essential to select the drug of choice due to the development of multidrug-resistant strains [14].

The emergence of multidrug-resistant Gram- negative rods is mentioned by different studies. It is usually associated with bad prognosis and medication-failure [15]. The antimicrobial resistance in *E*. *coli* is mainly attributed to the ESBLs; which could destroy various β-lactam antimicrobial agents as penicillins, various generations of cephalosporins, and carbapenems. [16].

*E*. *coli* have many virulence-associated factors, including adhesins, toxins, iron acquisition factors, lipopolysaccharides, polysaccharide capsules, and invasins, which are usually encoded on pathogenicity islands (PAIs), plasmids, and other mobile genetic elements. These factors are important in the epidemiology and pathophysiology of *E*. *coli* infections [17, 18]. Many virulence factors have been demonstrated to play variety of roles in the pathogenesis of Salmonella infections. These factors included flagella, capsule, plasmids, adhesion systems, and type 3 secretion systems (T3SS) encoded on the Salmonella pathogenicity island (SPI)-1 and SPI-2 and other SPIs [19].

Evaluation of meat at the retail level is recognized as a useful way to assess the risk of consumer exposure to enteric pathogens and antimicrobial resistance. During meat handling processes, meat may become contaminated with bacteria from the animal’s digestive tract, even under conditions of strict hygiene [20]. However, reports about *salmonella* species and ESBLs producing *Escherichia coli* isolates from raw cattle meat at butcher houses in Hawassa city are inadequate. Hence the current study aimed to assess the prevalence and antimicrobial susceptibility of *Salmonella* species and Extended-spectrum β-lactamase producing *Escherichia coli* from raw cattle meat at butcher houses in Hawassa city, Sidama regional state, Ethiopia.

## Materials and methods

### Study design, area and period

A cross-sectional study design was conducted at Hawassa city which is located 275 Km South of Addis Ababa, the capital city of Ethiopia, from September to December 2020.

### Ethics approval

Ethical approval was obtained from the Institutional Review Board of Hawassa University College of Medicine and Health Science. Participation was fully voluntary and written consent was obtained from each participant. Strict confidentiality was kept during data collection and report writing.

### Sample size and sampling technique

The sample size was calculated by using sample size determination for estimation of single population proportion formula. By using the anticipated population proportion of 20.8% [21], a study from Tigray, Northern Ethiopia, 95% confidence interval (z = 1.96) and 5% marginal error (d = 0.05), and assuming 10% non–response rate, the final sample size was 278. A convenient sampling technique was applied to select 278 butcher houses for interview and sample collection. From each selected butcher house 278 meat samples and 278 swabs were collected from knives and cutting boards.

### Laboratory sample collection

A total of 278 meat samples and 278 swabs from knives and cutting boards were collected from September 2020 to December 2020. Twenty five gram (25 g) of meat sample was minced and diluted into 225 ml of buffered peptone water. The swab samples from knives and cutting boards were put into a sterile test tube and suspended into a test tube containing 9 ml sterile buffered peptone water (BPW). All samples were labeled and packed in sterile plastic bags and transported to Hawassa University Teaching Laboratory for microbiological testing. Minced raw meat samples were pre-enriched in an appropriate amount of buffered peptone water to produce high resuscitation rates for bacteria and promote intense growth [22].

### Isolation and identification of *E*. *coli* and *Salmonella* species

*Salmonella* species were isolated and identified according to the technique suggested by the international organization for standardization [23]. Xylose lysine deoxycholate (XLD) agar (Oxoid, UK), MacConkey agar (Oxoid, UK), Hektoen Enteric (HE) agar (Oxoid, UK) plates were used for plating out and identification. A loop full of inoculums from buffered peptone water suspension was inoculated into XLD, HE and MacConkey agar plates and incubated at 37°C for 24 hours. Isolation of *E*. *coli* was conducted following standard procedure [24]. Upon arrival to the laboratory, all pre-enriched buffered peptone water broth raw meat samples were consequently inoculated to MacConkey agar and incubated at 37°C overnight. Bacterial growth were subjected in to lactose fermenter and non-lactose fermenter and the lactose fermenter colony were sub-cultured and incubated at 37°C for 24 hours. Identification of S*almonella* species and *E*. *coli* was done using different biochemical tests (Oxoid, UK) including Triple sugar iron (TSI) agar, Methyl red, urease, indole, motility agar and citrate tests.

### Screening and confirmation of ESBL-producing isolates

The ESBL screening test was performed by standard disk diffusion method by using ceftazidime (30 μg) and cefotaxime (30 μg) disks. After adjusting 0.5 McFarland’s standard, the suspensions were inoculated onto Muller-Hinton agar with a sterile cotton swab, and then the above two antibiotics disks were placed on the inoculated plate and incubated at 37°C for 24 hours. Isolates with reduced susceptibility to cefotaxime (≤ 27 mm) and ceftazidime (≤ 22 mm) around the disks were suspected as ESBLs- producers as recommended by CLSI guidelines [25].

ESBL producing *E*.*coli* was confirmed by using double disk approximation or double disk synergy method. After inoculation of the suspension onto Muller-Hinton agar, a disk of amoxicillin + clavulanic acid (20/10 μg) was placed in the center of the plate and then the disks of cefotaxime (30 μg) and ceftazidime (30 μg) were placed at a distance of 20 mm from the central disk on the same plate and the plate was incubated at 37°C for 24 hours and observed for an enhancement of inhibition zone of the β-lactam drugs caused by the synergy of the clavulanate was confirmed as ESBLs-production [25].

### Antimicrobial susceptibility testing

Antimicrobial susceptibility tests were performed on Mueller-Hinton agar using the Kirby-Bauer disk diffusion method. Morphologically similar 2–4 bacterial colonies were mixed with sterile normal saline and turbidity was adjusted to match 0.5 McFarland standards. The surface of Mueller-Hinton agar plate was uniformly inoculated with the prepared dilution using a sterile cotton swab. The antibiotic discs were applied to the surface of the inoculated agar. After 18–24 hrs of incubation, the diameter of growth inhibition around the disks was measured and interpreted as sensitive, intermediate, or resistant according to Clinical and Laboratory Standards Institute [26]. The antimicrobials disks tested were cefotaxime (30 μg), Ceftriaxone (30 μg), ceftazidime (30 μg), amoxicillin-clavulanic acid (20/10 μg), gentamycin(10 μg), cotrimoxazole (30 μg), and tetracycline (30 μg).

### Quality assurance

The quality of data was assured by properly designed and pre-tested questionnaires. The questionnaires were pre-tested a week before data collection on 5% of butcher shops that were not included in the study. The data collectors were trained on how to collect the data properly. Sterility of culture media were checked by incubating 5% of the batch at 35°C overnight and observed for bacterial growth. Performance of culture media was checked by using reference strains ATCC 25922 and ATCC 35664 for *E*.*coli* and *salmonella* respectively.

### Data analysis

Data were entered, coded and analyzed using statistical package for social science (SPSS) version 23 (IBM-SPSS Inc., Chicago, IL, USA). Data were organized, summarized, and presented in descriptive statistical methods. Bivariate and multivariate analysis were used to determine association contamination rate and bacterial isolates were determined by calculating odds ratio and 95% confidence interval and P-value < 0.05 was considered as statistically significant. Finally the result was presented using tables.

## Results

### Socio-demographic, sanitary and hygienic characteristics

During the study period, 278 meat handlers who sell cattle meat for communities and restaurants were enrolled. Out of these participants, 11(4%) were females and 267(96%) were males. The mean age was 25.8 years (standard deviation (SD), 5.1; range, 19–45 years), and the majority of them (54.4%) were in the age category of 25–35 years. Most of participants (40%) completed primary school level and the service year of the majority of participants (66.5%) was 2–5 years (Table 1).

**Table 1:** Socio-demographic, sanitary and hygienic characteristics of meat handlers in butcher shops at Hawassa city, Sidama regional state, Ethiopia, 2020 (n = 278).

Variables	Category	Frequency	Percent (%)
Sex	Male	267	96
	Female	11	4
Age(year)	< 25	119	42.8
	25–35	151	54.3
	36–45	8	2.9
Education status	No formal education	9	3.2
	Read and write	66	23.7
	1–7 grade	113	40.6
	8–12 grade	88	31.6
	Diploma & above	2	1
Service year	<1	44	15.8
	2–5	185	66.5
	6–10	46	16.5
	>10	3	1.2
Hand washing practice	Yes	128	44
	No	150	56
Medical screening status	Yes	180	64.7
	No	98	35.3
Appropriate outer protective coat	Yes	244	87.8
	No	34	12.2
Hair cover	Yes	84	30.2
	No	194	69.8
Meat safety training	Yes	226	81.3
	No	52	18.7
Use of Apron	Yes	25	9
	No	253	91
Use of glove while touching meat	Yes	27	9.7
	No	251	90.3
Touching Birr* while selling meat	Yes	117	42.1
	No	161	57.9
Cleaning equipment after work	Yes	190	68.3
	No	88	31.7
Wear jewelers	Yes	59	21.2
	No	219	78.8
Frequently cleaning meat storage area	Yes	274	98.6
	No	4	1.4

*Birr = Ethiopian unit of currency

Out of 278 meat handlers interviewed, 156 (56%) do not wash their hands regularly, 194(70%) do not cover their hair and 117 (42.1%) touch birr while selling meat, 180 (64.7%) were screened for medical status and 88 (31.7%) do not clean equipment after work. In almost all shops there was no wash-hand basins available (Table1).

### Prevalence of *Salmonella* species and ESBL producing *E*.*coli* and associated factors

Out of 556 raw meat and swab samples collected and analyzed, the overall prevalence for both *Salmonella* species and ESBL producing *E*.*coli* was 36 (6.5%). The prevalence of *Salmonella* and ESBL producing *E*.*coli* was 23(4.1%) and 13 (2.3%) respectively (Table 2).

**Table 2:** Prevalence of *Salmonella* species and ESBL producing *E*.*coli* from raw meat and swab samples collected from butcher shops at Hawassa city Sidama regional state, Ethiopia, 2020 (n = 556).

Isolates	Minced Meat		Swab		Total	
	n (%)		n (%)		n (%)	
	Yes	No	Yes	No	Yes	No
ESBL producing *E*.*coli*	7(2.5)	271(97.5)	6 (2.2)	272(97.8)	13(2.3)	543(97.7)
*Salmonella* species	6(2.2)	272(97.8)	17(6.1)	261(93.9)	23(4.1)	533(95.9)
Total	13(2.3)	543(97.7)	23(4.1)	533(95.9)	36(6.5)	520(93.5)

The bivariate and multivariable logistic regression analyses were done to assess the association between socio-demographic, sanitary and hygienic characteristics with culture positive minced meat and swab samples collected from butcher shops. In bivariate analysis, the variables hand washing practice (COR = 2.208, 95% CI, 1.249–3.904, P = 0.006) and touching birr (COR = .75, 95% CI, .433–1.299, P = 0.001) were candidate variables for multivariate analysis with p-value < 0.250, and hand washing practice and touching Birr while selling meat were statically significant associated risk factors with culture positive minced meat and swab samples (Table 3).

**Table 3:** Bivariate and multivariate analysis of factors associated with the prevalence of *Salmonella* species and ESBL producing *E*.*coli* from raw meat and swab samples collected from butcher shops at Hawassa city, Sidama regional state, Ethiopia, 2020 (n = 556).

Variables		Growth		COR(95%CI)	p-value	AOR(95%CI)	P-value
		*Yes (%)	No (%)				
Sex	Male	70(26.2)	197(73.8)	0.948(0.245–3.672)	0.938		
	Female	3(27.3)	8(72.7)	1			
Age(year)	< 25	33(27.7)	86(72.3)	0.869(0.167–4.523)	0.867		
	25–35	38(25.2)	113(74.8)	0.991(0.192–5.12)	0.991		
	36–45	2(25)	6(75)	1			
Educational status	No formal education	2(22.2)	7(77.2)	3.5(0.145–84.94)	0.441	.	
	1–8 grade	41	147	3.125(0.185–52.87)	0.43		
	9–12 grade	29(33)	59(67)	3.52(0.213–58.302)	0.38		
	Diploma & above	1(50)	1(50)	2.034(0.123–33.7)	0.62		
Hand washing practice	Yes	22(30.1)	51(69.9)	2.208(1.249–3.904)	0.006	2.434(1.358–4.365)	0.003
	No	100(48.8)	105(51.2	1			
Medical screening	Yes	48(26.6)	132(73.3)	1.062(0.606–1.862)	0.834		
	No	25(25.5)	73(74.5)	1			
Use of protective cloth	Yes	66(27)	178(73)	0.699(0.291–1.682)	0.424		
	No	7(20.6)	27(79.4)	1			
Hair cover	Yes	21(25)	63(75)	1.099(0.611–1.976)	0.754		
	No	52(26.8)	142(73.2)	1			
Meat safety training	Yes	61(27)	12(16)	0.811(0.399–1.649)	0.564		
	No	165(23)	40(19.)	1			
Use of Apron	Yes	8(32)	17(68)	0.735(0.303–1.783)	0.495		
	No	65(25.7)	188(74.3)	1			
Use of glove	Yes	7(25.9)	20(74.1)	1.019(0.412–2.521)	0.967		
	No	66(26.3)	185(73.7)	1			
Touching birr	Yes	27(23.1)	90(76.9)	0.75(0.433–1.299)	0.001	0.454(0.26–0.793)	0.006
	No	46(2.6)	115(71.4)	1			
Cleaning equipment after work	Yes	46(63)	27(37)	1.323(0.756–2.317)	0.327		
	No	142(69.3)	63(30.7)				
Wear jewelers	Yes	14(23.7)	47(76.3)	1.185(0.607–2.316)	0.619		
	No	59(26.9)	160(73.1)	1			
cleaning meat storage area	Yes	71(25.9)	203(74.1)	1.323(0.756–2.316)	0.298		
	No	2(50)	2(50)				

* Yes = growth at least in one of the two sample types collected from each butcher shop, CI = Confidence Interval, OR = Odds Ratio

### Antimicrobial susceptibility pattern

*Salmonella* isolates were highly sensitive to gentamycin 23 (100%), cotrimoxazole 20 (87%), ceftazidime 21 (91%), tetracycline 17 (74%) and cefoxitime 18 (78%) while resistant to amoxicillin/clavulanic acid15 (65%). ESBL producing *E*.*coli* isolates were highly sensitive to cotrimoxazole 13 (100%), gentamycin 12 (98.2%) while the isolates were resistant to cefotaximeand ceftazidime 13 (100%) for each (Table 4).

**Table 4:** Antibiotic susceptibility patterns of bacterial isolates from raw meat and swab samples collected from butcher shops at Hawassa city, Sidama regional state, Ethiopia, 2020 (n = 36).

Isolates Antibiotics (%)							
	Pattern	AMC	CTX	CTZ	COT	GN	TC
*Salmonella* spp.(n = 23)	S	6(26%)	18(78%)	21(91%)	20(87%)	23(100%)	17(74%)
	I	2(8.6%)	3(13%)	1(4.35%)	1(4.35%)	0	0
	R	15(65%)	2(8.6%)	1(4.35%)	2(8.6%)	0	6(26%)
ESBL producing *E*.*coli* (N = 13)	S	5(38%)	0	0	13(100%)	12(98.2%)	12(98.2%)
	I	1(7.69%)	0	0	0	1(1.2%)	1(1.2%)
	R	7(53.85%)	13(100%)	13(100%)	0	0	0
**Total (36)**	S	11(30.5%)	18(50%)	21(58.3%)	33(91.6%)	35(97.2%)	29(75%)
	I	3(8.3%)	3(8.3%)	1(2.7%)	1(2.7%)	1(2.7%)	1(2.7%)
	R	22(61.1%)	15(41.6%)	14(38.9%)	2(5.5%)	0	6(16.6%)

AMC—amoxicillin/clavulanic acid, CTX—cefotaxime, CTZ—ceftazidime, COT—cotrimoxazoleGN—gentamicine, TC—tetracycline, S- susceptible, I- intermediate, R- resistant, spp.- species.

The overall multidrug resistance (MDR) bacteria were 12/36 (33.3%). MDR isolates were 4 (17.4%) and 8 (61.5%) for *Salmonella* spp. and ESBL producing *E*.*coli* respectively (Table 5).

**Table 5:** Multi-drug resistant rates of bacterial isolates from raw meat and swab samples collected from butcher shops at Hawassa city, Sidama regional state, Ethiopia, 2020 (n = 36).

Isolate	R0 (%)	R1 (%)	R2 (%)	R3 (%)	≥R4 (%)	Total (%)
*Salmonella* spp.(n = 23)	5(21.7)	10(43.5)	4(17.4)	4(17.4)	0	4(17.4)
ESBL producing *E*.*coli* (N = 13)	0	0	5(38.4)	7(53.8)	1(7.7)	8(61.5)
Total (N = 36)	5(13.9)	10(27.8)	9(25)	11(30.5)	1(2.7)	12(33.3)

Key: R0- no antibiotic resistance, R1- resistance to one, R2-resistance to two, R3-resistance to three, R4-resistance to four and more than four antibiotics families

## Discussion

At the butcher houses, meat contamination might occur due to different potential factors such as storing food in dirty utensils, holding meat at a temperature that would allow microbial growth, poor hand washing practice, touching birr while selling meat, using unclean wrapping materials and due to lack of facilities for waste disposal. Moreover, the lack of awareness in basic personal cleanliness and safe meat handling increase the contamination of cattle meat by microorganisms [27].

This study revealed that the overall prevalence for both *Salmonella* species and ESBL producing *E*.*coli* was 36 (6.47%). The prevalence of *Salmonella* and ESBL producing *E*.*coli* was 23 (4.1%) and 13 (2.3%) respectively. The overall prevalence of both isolates in the raw minced meat samples was 13(36.1%) which had a lower proportion as compared with knife and chopping board swab samples, 23(63.9%). A study from Indonesia also showed that 58% of swab samples analyzed were contaminated with *E*. *coli* [28]. The contamination of knives and cutting boards is common because there is frequent contact with meat during selling, no regular washing of knives and cutting boards and no regular inspections. This might be posing great problems in a region where eating raw meat is a common tradition.

In this study, the prevalence of ESBL producing *E*.*coli* (2.3%) % is lower than the study finding in South Korea 20% [29] and in Tanzania 4.7% [30]. In addition to the above methodological differences, the observed differences might be also related to the abuse of third generation cephalosporins in food animals, because the use of these antibiotics is greatly linked to the recent emergence of ESBLs-producing bacteria. The prevalence of *Salmonella* species (4.1%), is higher than that of studies conducted in Dire Dawa (2.75%) [31] and Addis Abeba (3.7%) [32]. This variation might be due to the differences in the hygienic and sanitary practices in the butcher shops especially poor hand washing practices. Studies conducted in Malaysia [7], Ghana [33] and Jimma [34] showed the prevalence of *salmonella* species to be 10%, 30%, and 11.3% respectively which were higher than the current study. This difference might be due to hygiene and sanitation practice, methodology differences in sample collection and processing procedure.

Among meat handlers, 56% of them do not wash their hands regularly, which is higher than the study conducted in Bishoftu 9.4% [35]. This difference might be due to a lack of awareness of meat handlers on personal hygiene. Poor hand washing practice has a significant association with the prevalence of *salmonella* and *E*.*coli* in this study.

The current study showed that both isolates were highly sensitive to gentamicin, cotrimoxazole, ceftazidime, tetracycline and cefotaxime (85–100%) and (50–65%) of the isolates were resistant to amoxicillin-clavulanic acid. A study conducted in Nepal showed that higher resistance to amoxicillin- clavulnic acid (100%) and tetracycline (93%) [4] which is higher than the current study Another study conducted in Mekelle revealed 16(20.5%) multi drug resistance isolates which is lower than the current study (36%) [21]. This variation might be due to differences in sample sizes, characteristics of the antibiotics, the existence of a different strain of the bacteria, increasing of the resistant gene. Compared to other studies, the current study showed a low level of drug resistance which might be related to the fewer antimicrobial panels we used and the overall multidrug resistance bacteria were (36%).

### Limitations of the study

PCR based detection and gene sequencing of the antimicrobial resistance genes (ESBLs genes) in the multidrug resistant strains were not performed because it was not applicable since our laboratory has no facility for molecular analysis.

## Conclusion

In the present study, the overall prevalence of both isolates was 36 (6.5%) while the prevalence of *Salmonella* and ESBL producing *E*.*coli* was 23(4.1%) and 13(2.3%) respectively. The overall multidrug resistance bacteria were (33.3%). The presence of ESBLs-producing strains may have contributed for the occurrence of multidrug resistant isolates to many classes of antibiotics. Furthermore, unhygienic practices of meat handlers observed may have contribution for contaminations of meat with *E*. *coli* isolates. This suggested that the necessity of routine screening of ESBL is crucial for the early detection and treatment. Hence, policies should be planned and applied to improve the knowledge and practice of butchers about handling and processing of meat.

## Supporting information

S1 Questionnaire (DOCX)
