# Supplementary material for: High prevalence and antimicrobial susceptibility pattern of salmonella species and extended-spectrum β-lactamase producing Escherichia coli from raw cattle meat at butcher houses in Hawassa city, Sidama regional state, Ethiopia
Source: PLoS One. 2022 Jan 14;17(1):e0262308. doi: 10.1371/journal.pone.0262308 (PMC8759633; doi:10.1371/journal.pone.0262308)
Supplement: S1 Questionnaire — (DOCX) [file pone.0262308.s001.docx]

**Questionnaire**

High prevalence and antimicrobial susceptibility pattern of *Salmonella* species and extended-spectrum β-lactamase producing *Escherichia coli* from raw cattle meat at butcher houses in Hawassa city, Sidama regional state, Ethiopia

Participant Identification (Meat handler identification)

1. **Socio-demography**
2. Full name: ____________ Code_________
3. Sex:
   1. Female
   2. Male
4. Age (in years): _________
5. Educational status:
   1. No formal education
   2. Read and write
   3. 1-6 grade
   4. 7-12 grade
   5. Graduate
6. Year/s of service:
   1. <1 Year
   2. 2-5 Years
   3. 6-10 Years
   4. >10 Years
7. **Hygiene and sanitation**
8. Hand-washing practice before touching meat:
   1. Yes
   2. No
9. For question number 6, If the answer is yes, is it:
   1. with soap
   2. without soap
10. Medical screening status:
    1. Yes
    2. No
11. Appropriate outer protective coat
    1. Yes
    2. No
12. Hair covers
    1. Yes
    2. No
13. Hygiene training
14. Yes
15. No
16. Use of apron
    1. Yes
    2. No
17. Use of glove while touching meat
    1. Yes
    2. No
18. Touching birr with bare hand during selling of meat
    1. Yes
    2. No
19. Cleaning equipment after work
    1. Yes
    2. No

**መጠይቅ (Questionnaire-Amharic version)**

በሀዋሳ ከተማ አስተዳደር ስር የሚገኙ የከብቶች ስጋ አቅርቦት ላይ ከተሰማሩ የንግድ ተቋማት ስጋ በመውሰድ ኢ. ኮላይ እና የሳልሞኔላ ባክቴሪያዎችን መለየትና ባክቴሪያዎቹ ያላቸውን የተለያዩ መድሃኒቶችን የመቋቋም አቅማቸውን ማወቅ

**የተሳታፊ መለያ**

**I ሶሺዮ-ዲሞግራፊ**

1. ሙሉ ስም_____________________ ኮድ ________
2. ጾታ
   1. ሴት
   2. ወንድ
3. ዕድሜ (በአመታት) ___________
4. የትምህርት ደረጃ፡-
   1. መደበኛ ትምህርት የለም
   2. ማንበብ እና መጻፍ
   3. 1-6 ክፍል
   4. 7-12 ክፍል
   5. ተመራቂ
5. የአገልግሎት ዓመታት
   1. <1 አመት
   2. 2-5 ዓመታት
   3. 6-10 ዓመታት
   4. > 10 ዓመታት

**II የንፅህና አጠባበቅ**

1. ስጋ ከመንካት በፊት እጅን የመታጠብ ልምድ፡-
   1. አዎ
   2. አይ
2. ለጥያቄ ቁጥር 6 መልሱ አዎ ከሆነ፡-
   1. በሳሙና
   2. ያለ ሳሙና
3. የሕክምና የማጣሪያ ሁኔታ፡-
   1. አዎ
   2. አይ
4. ተስማሚ የውጭ መከላከያ ካፖርት
   1. አዎ
   2. አይ
5. የፀጉር ሽፋን
   1. አዎ
   2. አይ
6. የንጽህና ስልጠና
   1. አዎ
   2. አይ
7. የአፕሮን አጠቃቀም
   1. አዎ
   2. አይ
8. ስጋን በሚነኩበት ጊዜ ጓንት መጠቀም
   1. አዎ
   2. አይ
9. ስጋ ሲሸጥ በባዶ እጅ ብር መንካት
   1. አዎ
   2. አይ
10. ከስራ በኋላ እቃዎች ማጽዳት
    1. አዎ
    2. አይ
